# The Role of Glutamine Oxoglutarate Aminotransferase and Glutamate Dehydrogenase in Nitrogen Metabolism in *Mycobacterium bovis* BCG

**DOI:** 10.1371/journal.pone.0084452

**Published:** 2013-12-19

**Authors:** Albertus J. Viljoen, Catriona J. Kirsten, Bienyameen Baker, Paul D. van Helden, Ian J. F. Wiid

**Affiliations:** DST/NRF Centre of Excellence for Biomedical Tuberculosis Research, MRC Centre for Molecular and Cellular Biology, Division of Molecular Biology and Human Genetics, Faculty of Medicine and Health Sciences, Stellenbosch University, Tygerberg, Cape Town, South Africa; Colorado State University, United States of America

## Abstract

Recent evidence suggests that the regulation of intracellular glutamate levels could play an important role in the ability of pathogenic slow-growing mycobacteria to grow *in vivo*. However, little is known about the *in vitro* requirement for the enzymes which catalyse glutamate production and degradation in the slow-growing mycobacteria, namely; glutamine oxoglutarate aminotransferase (GOGAT) and glutamate dehydrogenase (GDH), respectively. We report that allelic replacement of the *Mycobacterium bovis* BCG *gltBD-*operon encoding for the large (*gltB*) and small (*gltD*) subunits of GOGAT with a hygromycin resistance cassette resulted in glutamate auxotrophy and that deletion of the GDH encoding-gene (*gdh*) led to a marked growth deficiency in the presence of L-glutamate as a sole nitrogen source as well as reduction in growth when cultured in an excess of L-asparagine.

## Introduction

**Figure 1:**
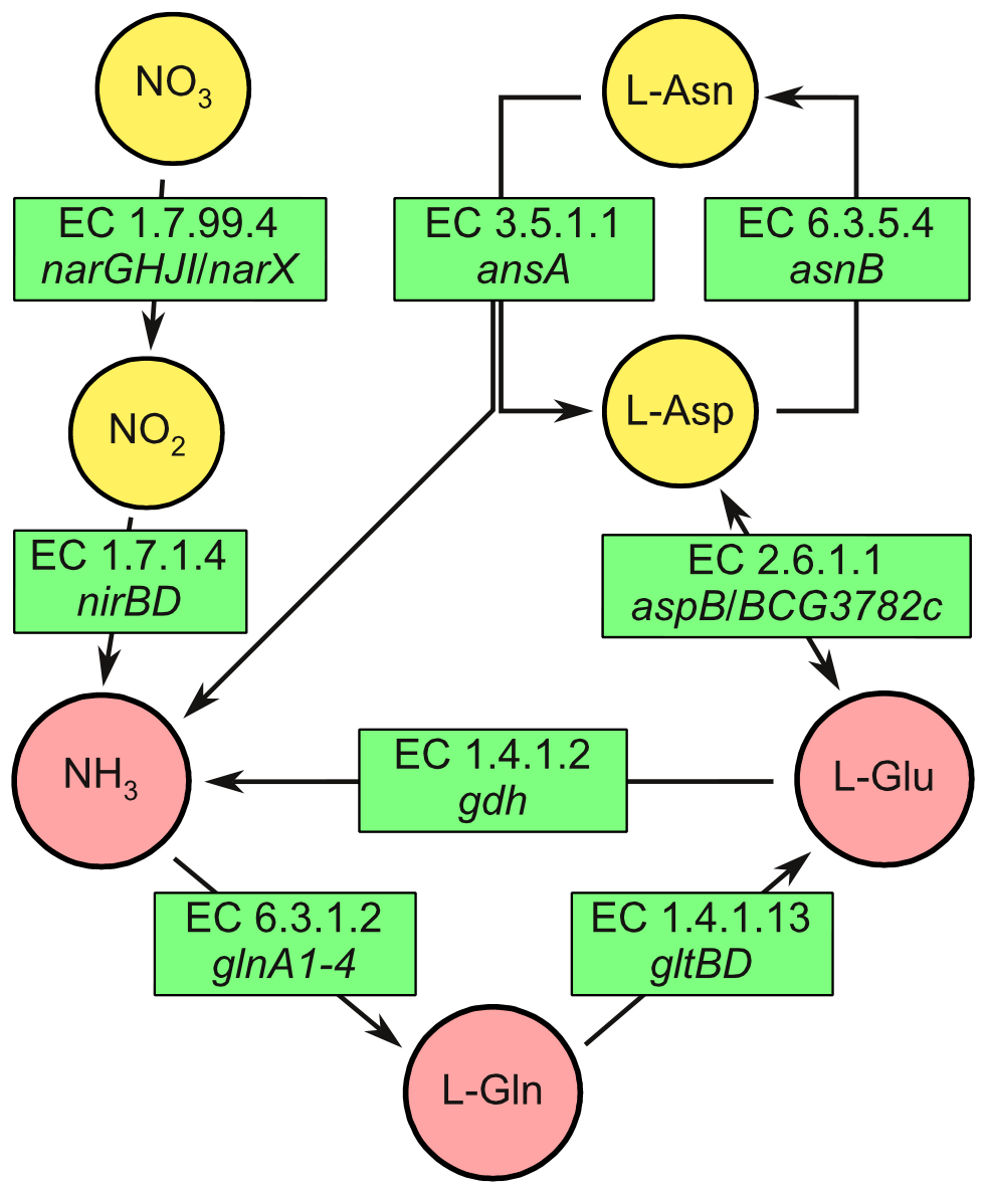
Genes involved in nitrogen metabolism in the slow growing mycobacterium *M. bovis* BCG. Ammonia is assimilated in the production of L-glutamine and L-glutamate. Together, L-glutamine, L-glutamate, and L-aspartate act as precursors or nitrogen donors to most other nitrogenous compounds in the mycobacterium. The map was constructed from the combined PATRIC pathways for *M. bovis* BCG *str*. Pasteur 1743P2 nitrogen metabolism and alanine, aspartate and glutamate metabolism [3]. Genes were assigned to the EC numbers by PATRIC and/or Refseq and/or Legacy BRC.

The nitrogen metabolic pathways of pathogenic mycobacteria are factors which allow the bacteria to survive and replicate in host cells [1,2]. These pathways may be a potential source of novel target molecules that could be exploited in future drug development. Central nitrogen metabolism in slow growing mycobacteria mainly involves the biochemical pathways that fix inorganic ammonium and produce glutamine, glutamate and aspartate (Figure 1) [3]. These three amino acids act as precursors or nitrogen donors in the production of nearly all other nitrogenous molecules in the cell.

Glutamine synthetase (GS), which assimilates inorganic ammonium for the production of glutamine, has been studied extensively in *Mycobacterium tuberculosis* and related mycobacteria. The gene *glnA1*, which encodes the major isoform of GS in *M. tuberculosis* was shown to be essential for virulence in guinea pigs [2]. GS activity in *M. tuberculosis* is regulated at both the transcriptional and post-translational level. The latter is achieved by an adenylyl transferase encoded by the gene *glnE* [4–7]. It has been shown that a *glnE* deletion mutant of *M. tuberculosis* is only viable when GS is inhibited by methionine sulfoximine and the culture supplemented with glutamine [6]. Furthermore, the serine threonine protein kinase G (PknG) which is involved in the homeostatic regulation of glutamine and glutamate levels has been shown to be important to *in vivo* growth of *M. tuberculosis* [8]. This indicates that central nitrogen metabolism is tightly regulated in *M. tuberculosis*. Although an interaction between GlnE and PknG in the control of glutamine metabolism was suggested, it has not been investigated [8]. It was found, however, that PknG phosphorylates glycogen accumulation regulator A (GarA), thereby modulating its interaction with the glutamate producing enzyme, glutamate oxoglutarate aminotransferase (GOGAT) and the glutamate catabolizing enzyme, glutamate dehydrogenase (GDH) [9,10]. Phosphorylation of GarA at residue threonine 21 by PknG is thought to abrogate both its inhibition of GDH activity and its stimulation of GOGAT activity, leading to a decrease in glutamate levels [10]. These results suggest that the tight regulation of glutamate levels might be important to the survival and proliferation of *M. tuberculosis* during infection of the host.

Although it was found by Himar-1 based transposon mutagenesis that the genes *gltB* (encodes for the large subunit of GOGAT), *gltD* (encodes for the small subunit of GOGAT) and *gdh* are essential to the *in vitro* growth of *M. tuberculosis* [1,11,12], we could disrupt *gltBD* (*BCG*_*3922c*-*BCG*_*3921c*) and *gdh* (*BCG_2496c*) in *Mycobacterium bovis* BCG, a closely related slow growing mycobacterium. *M. bovis* BCG is considered to be non-pathogenic to humans, but it does survive in macrophages and maintains a degree of virulence [13]. We show that while GOGAT is required for the *de novo* synthesis of glutamate, GDH is important for the utilization of glutamate as a sole nitrogen source and for growth with high levels of asparagine in the culture medium even in combination with glutamate and/or ammonium.

## Materials and Methods

### Growth of bacteria

All bacterial strains used are listed in Table S1. *Escherichia coli* was cultured with shaking at 200 rpm in Lysogeny Broth (LB) and on LB agar at 37°C. *M. bovis* BCG was cultured without agitation in Difco Middlebrook 7H9 liquid medium (Becton Dickinson, USA) supplemented with 10% (v/v) ADC (50 g/L bovine serum albumin fraction v, 20 g/L D-glucose, 15 mg/L catalase), 0.2% (v/v) glycerol, and 0.05% (v/v) Tween 80 in 25 cm^2^ (5 - 10 ml) and 75 cm^2^ (30 ml) cell culture flasks (Nunc, Denmark) and on BBL 7H11 agar base (Becton Dickinson, USA) supplemented with 0.5% (v/v) glycerol and 10% (v/v) BBL Middlebook OADC (Becton Dickinson, USA). In order to investigate growth of bacteria in the presence of different nitrogen sources, we prepared modified Middlebrook 7H9 medium which lacked the nitrogen sources present in 7H9, namely L-glutamate, ammonium sulphate and ferric ammonium citrate (-N7H9; sodium citrate, 0.1 g/L; pyridoxine, 1 mg/L; biotin, 0.5 mg/L; disodium phosphate, 2.5 g/L; monopotassium phosphate, 1 g/L; ferric citrate, 40 mg/L; magnesium sulphate, 50 mg/L; CaCl_2_, 0.5 mg/L; ZnSO_4_, 1 mg/L; CuSO_4_, 1 mg/L; glycerol, 0.2% v/v; Tween80, 0.05% v/v; ADC, 10% v/v), which was subsequently supplemented with different nitrogen sources as indicated in the text. *M. bovis* BCG starter cultures maintained in 7H9 were washed twice with –N7H9 before they were used to inoculate –N7H9 medium supplemented with different nitrogen sources for growth curve determinations. Antibiotic concentrations used in *M. bovis* BCG cultures were as follows: hygromycin, 50 μg/ml on solid medium and 25 μg/ml in liquid medium; kanamycin, 20 μg/ml; and gentamycin, 2.5 μg/ml. Antibiotic concentrations used in *E. coli* cultures were: ampicillin, 50 μg/ml; hygromycin, 100 μg/ml; kanamycin, 50 μg/ml; and gentamicin, 5 μg/ml.

### Generation of *ΔgltBD::hyg* and *ΔgltBD::hyg attB*::pGCgltBD strains

All oligonucleotides and plasmids used are listed in Table S1. All molecular cloning procedures were carried out as described elsewhere [14]. The mycobacterial recombineering method developed by van Kessel et al. (2007) was used to replace the *gltBD* operon with a hygromycin cassette [15]. Briefly, the specific oligonucleotides UgltBDF and UgltBDR, harbouring SphI and NcoI restriction endonuclease recognition sites respectively, were used to generate a 499bp PCR fragment of the region directly upstream of the *gltBD* operon. The PCR fragment was cloned into pGEM-T Easy, which was subsequently digested with SphI and NcoI to obtain a restriction fragment of the upstream (U) region. The restriction fragment was directionally cloned into the SphI-NcoI sites of pMNFhyg to obtain pMNFhygU. The specific oligonucleotides DgltBDF and DgltBDR, harbouring SpeI and PstI restriction endonuclease recognition sites respectively, were used to generate a 511bp PCR fragment of the region directly downstream (D) of the *gltBD* operon. The PCR fragment was cloned into pGEM-T Easy, and subsequently digested with SpeI and PstI to obtain a restriction fragment of the D region. The restriction fragment was directionally cloned into the SphI*-*NcoI sites of pMNFhygU to produce pAVΔgltBD. The linear allelic exchange substrate was obtained by digesting pAVΔgltBD with SphI and PstI, and electro-transformed into *M. bovis* BCG carrying the pJV53 recombineering plasmid (wt-BCG pJV53) as described previously [15]. Before electro-transformation, wt-BCG pJV53 was cultured in 7H9 supplemented with 0.05% Tween 80 and 0.2% (w/v) succinate to a density of approximately OD_600_ = 0.5 at which point acetamide was added to a final concentration of 0.2% (w/v). The culture was incubated overnight at 37°C without agitation and used to prepare electrocompetent cells as described previously [16,17]. Hygromycin resistant colonies were subjected to PCR and Southern blot analysis as described elsewhere (see section on Southern blot analysis). The *ΔgltBD::hyg* mutant strain (from here on referred to as *ΔgltBD*) was complemented with a functional *gltBD* operon using the integrating vector pGINTO [18]. Briefly, the *gltBD* operon, along with a 525bp region upstream of the gene was amplified by PCR using Phusion High Fidelity PCR polymerase (FinnZymes, Finland) and the specific oligonucleotides CgltBDF and CgltBDR. The CgltBD fragment was ligated to pGINTO that was linearized with ScaI to produce pGCgltBD. Electro-competent *ΔgltBD* bacteria were transformed with pGCgltBD. Complemented colonies (referred to as *ΔgltBD attB*::pGCgltBD) were gentamicin resistant. The progenitor strain of the *ΔgltBD* mutant, wt-BCG pJV53, was included in analyses (as indicated in the text) to control for residual effects of the recombineering plasmid. All fragments generated by PCR within pAVΔgltBD and pGCgltBD were subjected to DNA sequencing (Stellenbosch University Central Analytical Facility) to confirm that no mutations were introduced by the polymerases.

### Generation of *Δgdh* and *Δgdh attB*::pGCgdh strains

**Figure 2:**
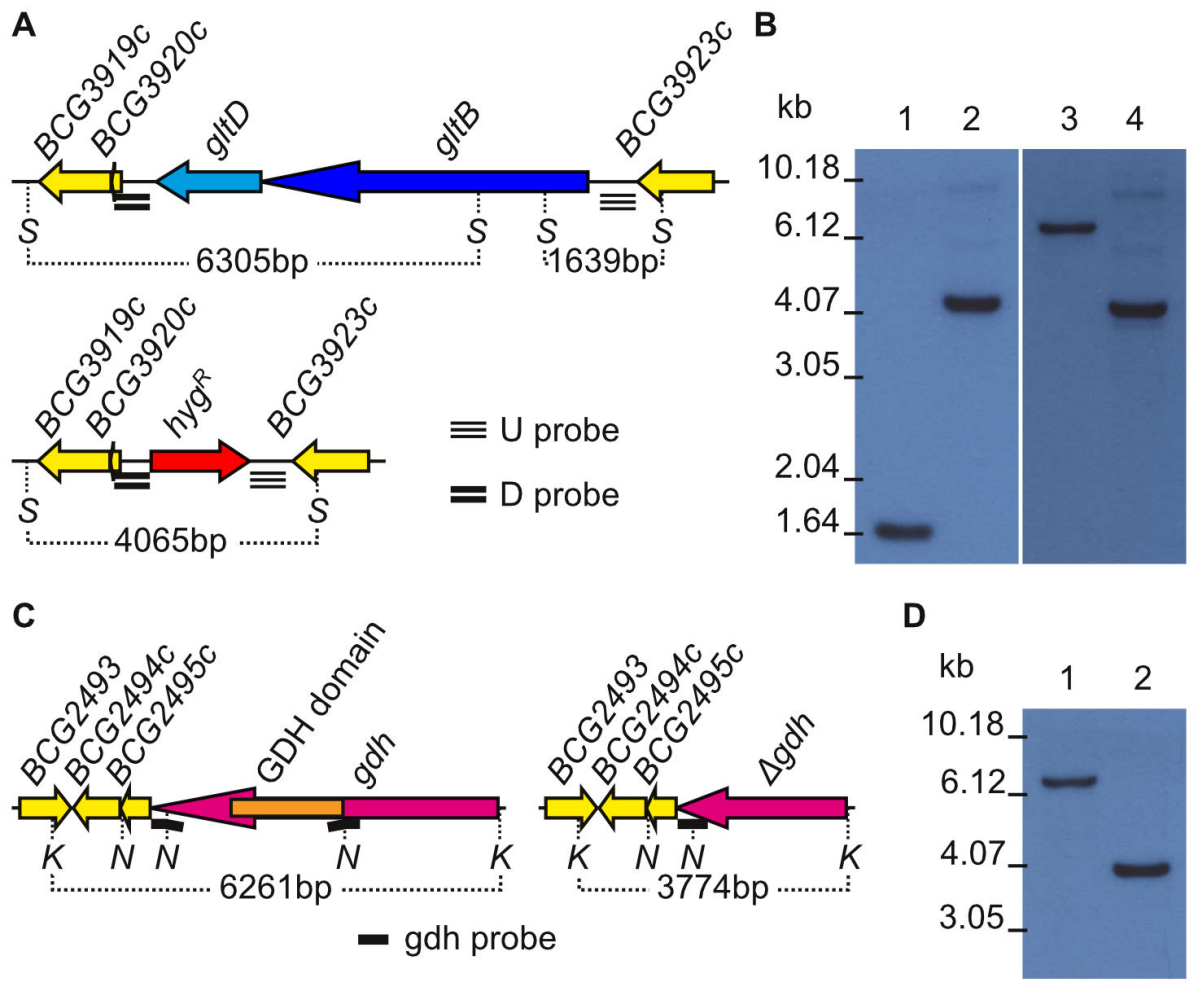
Replacement of the *gltBD* operon with a hygromycin cassette and gene deletion of *gdh*. A) Comparison of the wild-type *M. bovis* BCG and mutant *gltBD* regions. B) Southern blot analysis of wild-type *M. bovis* BCG (lane 1) and *ΔgltBD::hyg* (lane 2) with a probe that hybridises upstream of *gltB*. Southern blot analysis of wild-type *M. bovis* BCG (lane 3) and *ΔgltBD::hyg* (lane 4) with a probe that hybridises downstream of *gltD*. C) Comparison of the wild-type *M. bovis* BCG and mutant *gdh* regions. The GDH domain region is the sequence in *gdh* which aligned with a 98% query coverage (66% identity, 80% positives) in a blastp to the GDH domain sequence of the previously characterised Streptomyces *clavuligerus* L-180 GDH [36]. The NruI fragment spanning the GDH domain is deleted in the *Δgdh* chromosome. The probe which is complementary to fragments both upstream and downstream of the GDH domain does not hybridise across its full length with wild-type *M. bovis* BCG DNA, but does with *Δgdh* mutant DNA, as indicated in the figure. D) Southern blot analysis of wild-type *M. bovis* BCG (lane 1) and *Δgdh* (lane 2). *S*, SphI; *hyg* ^*R*^, hygromycin resitance cassette; *K*, KpnI; *N*, NruI; U, upstream; D, downstream.

All oligonucleotides and plasmids used are listed in Table S1 and all molecular cloning procedures were carried out as described elsewhere [14]. The *gdh* gene was disrupted by allelic-exchange using the plasmids p2Nil and pGOAL17 [19]. Briefly, a fragment that spans the GDH domain of *gdh*, as well as approximately 1kb of the 5’ and 3’ sequences flanking the GDH domain, was amplified by PCR with long PCR enzyme mix (Fermentas, USA) using the specific oligonucleotides gdhF and gdhR. The gdh PCR amplicon was cloned into the pGEM-T Easy vector to generate pGEMgdh. The central in-frame NruI fragment spanning the GDH domain (see Figure 2A) was subsequently excised to obtain the vector pGEMΔgdh containing adjacent 5’ and 3’ flanking sequences of the GDH domain. The KpnI fragment containing the GDH domain flanking regions was excised from pGEMΔgdh and cloned into the KpnI site of p2Nil to produce p2NilΔgdh. Finally, a PacI cassette containing the genes *sacB* and *lacZ* from pGOAL17 [19] was excised and cloned into the PacI site of p2NilΔgdh to produce pAVΔgdh. The pAVΔgdh deletion construct was treated with 100 mJ UV irradiation prior to electro-transformation into *M. bovis* BCG as described previously [20]. An exponentially growing *M. bovis* BCG culture (OD_600_ 0.5-0.8) was made electro-competent and electro-transformed with pAVgdh (see section on generation of *ΔgltBD* strain). Bacteria in which pAVΔgdh was integrated into the chromosome by homologous recombination were resistant to kanamycin (kan^R^) and coloured blue in the presence of 50 μg/ml 5-bromo-4-chloro-indolyl-β-D-galactopyranoside (X-gal). Hence, blue kan^R^ colonies were transferred to liquid culture without antibiotics, grown to mid-exponential phase (OD_600_ = 0.5 - 0.8) and plated on 7H11 supplemented with 2% sucrose and 50 μg/ml X-gal. Bacteria in which pAVΔgdh was lost from the chromosome by a second homologous recombination event were resistant to sucrose and remained white in the presence of X-gal. White suc^R^ colonies were subjected to PCR and Southern blot analysis as described elsewhere (see section on Southern blot analysis). The *Δgdh* mutant strain was complemented with a functional *gdh* gene also using the integrating vector pGINTO (Table S1). Briefly, the *gdh* gene, along with a 565bp region upstream of the gene, was amplified by PCR using Phusion High Fidelity PCR polymerase (FinnZymes, Finland) and the specific oligonucleotides CgdhF and CgdhR (Table S1). The Cgdh8 fragment was ligated to pGINTO that was linearized with ScaI to produce pGCgdh (Table S1). Electro-competent *Δgdh* bacteria were transformed with pGCgdh. Complemented colonies (*Δgdh attB*::pGCgdh) were gentamicin resistant. All fragments generated by PCR within pAVΔgdh and pGCgdh were subjected to sequencing (Stellenbosch University Analytical Facility) to confirm that no mutations were introduced by the polymerases.

### Southern blot analysis

Genomic DNA (gDNA) was purified from wild type *M. bovis* BCG (wt-BCG), *ΔgltBD* and *Δgdh* strains as described previously [21] and digested (6 μg) with SphI (*ΔgltBD*, wt-BCG) and KpnI (*Δgdh*, wt-BCG) for 16 hours. The resultant restriction fragments were separated by gel electrophoresis (0.8% agarose), chemically denatured and transferred to a Hybond N1 membrane (Amersham) by Southern transfer for 20 hours as previously described [21]. DNA was heat fixed to the membrane at 80°C for 2 hours. Hybridisation and detection was performed with the Amersham ECL Nucleic acid Labelling kit according to the manufacturer’s recommendations. The PCR products obtained from UgltBDF and UgltBDR as well as DgltBDF and DgltBDR, were used as probes for the Southern blotting analysis of the *ΔgltBD* mutant. A probe for Southern blotting analysis of the *Δgdh* mutant was generated by PCR using the specific oligonucleotides PgdhF and PgdhR (Table S1), HotStarTaq polymerase (Qiagen) and the pGEMΔgdh construct as template.

### Statistical analysis

Statistical analyses were carried out with the statistics software GraphPad Prism version 5.01. All differences in colony forming unit (cfu) data for the growth curve analyses were evaluated as randomised block design experiments by two-way repeated measures ANOVA and Bonferroni post-tests. In each analysis, the matching of subjects (strains) between experimental repeats was efficient (p < 0.05). Probabilities of < 0.05 were considered significant.

## Results

### Generation of *ΔgltBD* and *Δgdh* strains

Putative *ΔgltBD colonies* and *Δgdh* colonies were initially screened by PCR (data not shown) before analysis by Southern blot (Figure 2). When Southern blot analysis was done with probes that hybridised in the region directly upstream of *gltB* (a 1639 bp SphI fragment spanning the start codon of the *gltBD* operon) or directly downstream of *gltD* (a 6305 bp SphI fragment which spans the 3’ portion of *gltB*, the entire *gltD* and two genes downstream of *gltD*) the corresponding fragments were observed for wt-BCG (Figure 2B). Neither of these fragments were observed for the *ΔgltBD* mutant (Figure 2B). However a single fragment corresponding to the expected 4065 bp was observed for the *ΔgltBD* mutant when either probe was used (Figure 2B). A fragment corresponding to the expected 6261 bp KpnI fragment spanning *gdh* was observed for wt-BCG, whereas a fragment corresponding to the 3774 bp KpnI fragment spanning *gdh*, containing a 2487 bp deletion, was detected for the *Δgdh* mutant (Figure 2D). Growth of the *ΔgltBD* mutant was markedly slower on both 7H11 and 7H10 (slower than on 7H11) than growth of wt-BCG or the complemented *ΔgltBD* strain (Figure S1). However, growth of the *Δgdh* mutant was comparable to that of wt-BCG on both 7H10 and 7H11.

### Growth of wt-BCG in 7H9 containing different nitrogen sources

**Figure 3:**
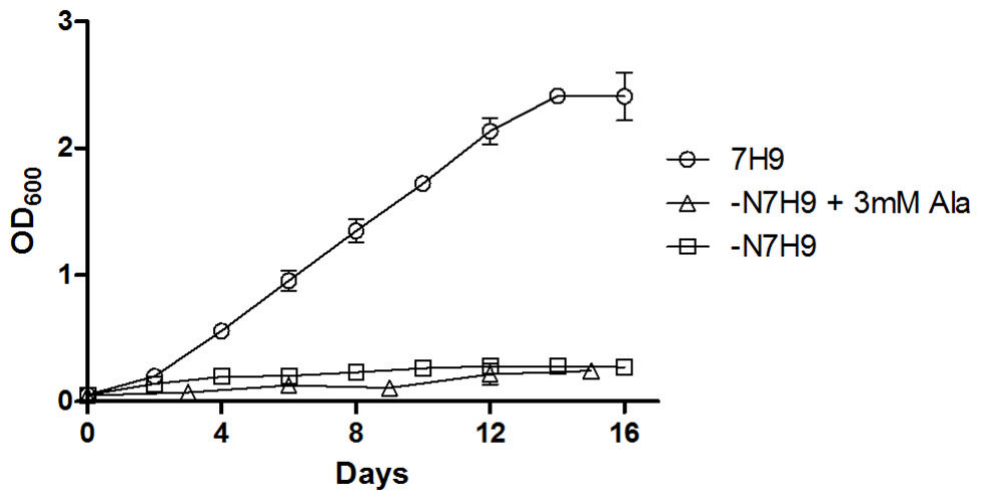
Growth of *M. bovis* BCG in standard 7H9, 7H9 lacking nitrogen sources (‑N7H9) and 7H9 containing alanine as sole nitrogen source (‑N7H9 + 3 mM L-Ala). Mean OD measurements with standard deviations were calculated with growth curve data obtained from three independent experiments performed for each condition tested.

In order to investigate the *in vitro* growth requirements of the *ΔgltBD* and *Δgdh* mutants, we modified both 7H9 (containing 3.4 mM L-Glu and 3.8 mM ammonium sulphate, see materials and methods) and ‑N7H9 (see materials and methods) by supplementation with different nitrogen sources (see Table S2). wt-BCG growth in all formulations was comparable to that in 7H9, except in the case of ‑N7H9 without nitrogen source supplementation (Figure 3, ) or with 3 mM L-Ala (Figure 3, ). This result is in line with a previous finding that *M. bovis* BCG cannot utilize alanine as a sole nitrogen source because of a frame-shift mutation in the gene that encodes for alanine dehydrogenase (*ald*) and inhibition of GS by non-catabolised alanine [22].

### *In vitro* requirement of *gltBD*

**Figure 4:**
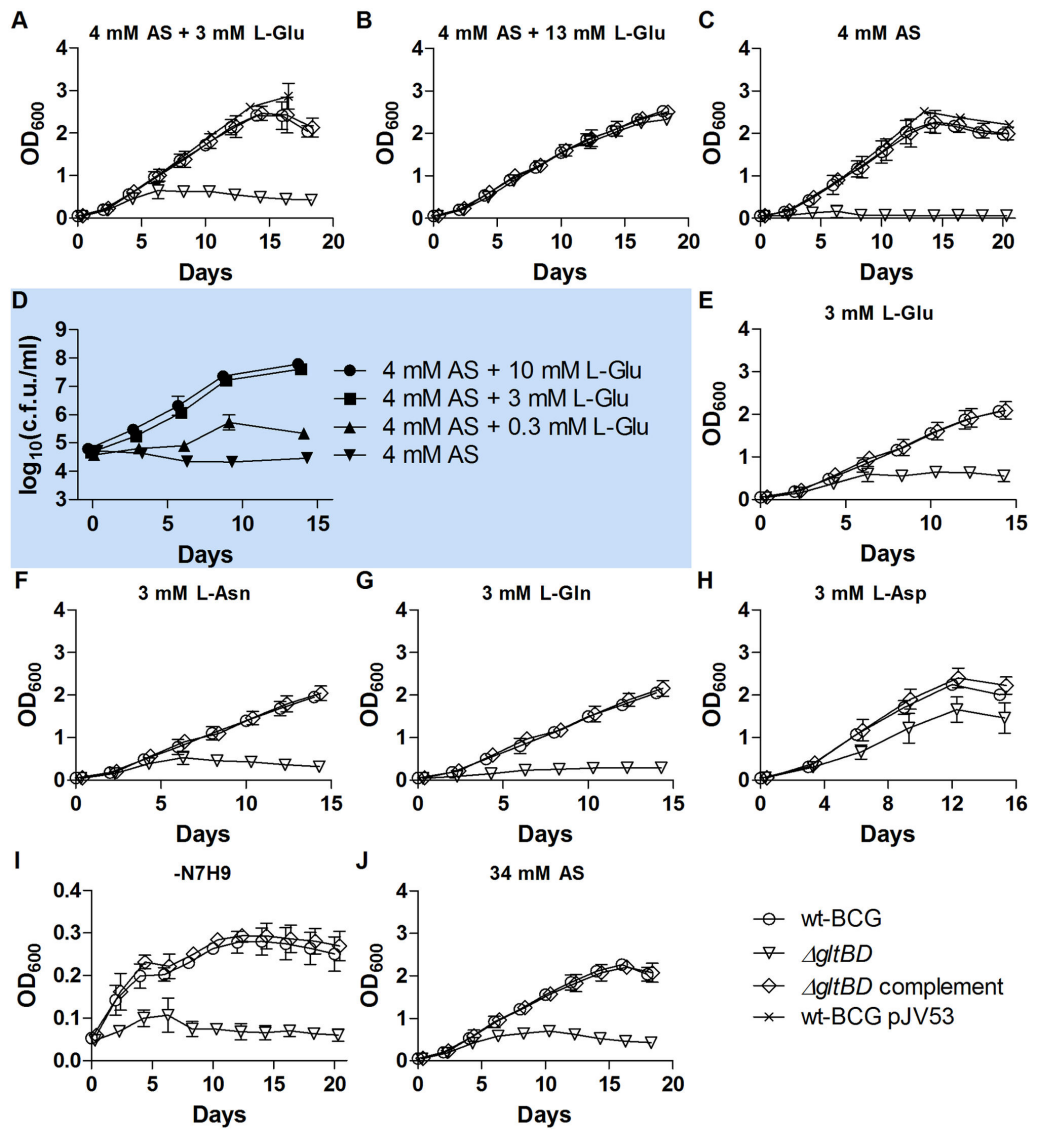
Growth of *ΔgltBD* in 7H9 with different nitrogen sources. Growth of wt-BCG, the *ΔgltBD* mutant and the *ΔgltBD* complemented strain in (A) standard 7H9 containing approximately 4 mM ammonium sulphate (AS, (NH_4_)_2_SO_4_) and 3 mM L-Glu, (B) 7H9 + 10 mM L-Glutamate, or (C) ‑N7H9 (nitrogen-depleted 7H9) + 4 mM AS. Growth of the *ΔgltBD* mutant in (D) ‑N7H9 + 4 mM AS supplemented with increasing concentrations of glutamate. Cultures for cfu/ml determinations were inoculated to OD_600_ = 0.0005 (cfu/ml of approximately 10^5^). Log_10_(cfu/ml) of *ΔgltBD* cultured in -N7H9 + 4 mM AS supplemented with 10 mM L-Glu was different from log_10_(cfu/ml) of *ΔgltBD* cultured in -N7H9 + 4 mM AS at every time point after and including 3 days (P < 0.001). Log_10_(cfu/ml) of *ΔgltBD* cultured in -N7H9 + 4 mM AS supplemented with 3 mM L-Glu was different from log_10_(cfu/ml) of *ΔgltBD* cultured in -N7H9 + 4 mM AS at 3 days (p < 0.01) and every following time point (p < 0.001). Log_10_(cfu/ml) of *ΔgltBD* cultured in -N7H9 + 4 mM AS supplemented with 0.3 mM L-Glu was different from log_10_(cfu/ml) of *ΔgltBD* cultured in -N7H9 + 4 mM AS at 6 days (p < 0.01) and every following time point (p < 0.001). Growth of wt-BCG, the *ΔgltBD* mutant and the *ΔgltBD* complement strain in (E) –N7H9 + 3 mM L-Glu, (F) –N7H9 + 3 mM L-Asn, (G) ‑N7H9 + 3 mM L-Gln, (H) –N7H9 + 3 mM L-Asp, (I) unmodified –N7H9, or (J) 7H9 + 30 mM AS. Mean OD measurements with standard deviations presented in panels A-C and E-J and mean log_10_(cfu/ml) with standard errors presented in panel D were calculated with growth curve data obtained from three independent experiments performed for each condition tested. In some instances error bars were smaller than the symbols used to depict the means. AS, ammonium sulphate.

The growth of the *ΔgltBD* mutant was markedly impaired in standard 7H9, which contains 3.4 mM L-Glu and 3.8 mM (NH_4_)_2_SO_4_ (Figure 4A, ), but was restored to wt-BCG levels when an additional 10 mM L-Glu was introduced in the medium (Figure 4B, ). Moreover, no growth of the *ΔgltBD* mutant was observed when ammonium was used as sole nitrogen source (Figure 4C, and D, ). Growth of the *ΔgltBD* mutant was comparable to that in 7H9 when L-Glu (Figure 4E, ) or L-Asn (Figure 4F, ), but not when L-Gln (Figure 4G, ) was added as sole nitrogen sources. In addition, growth of the *ΔgltBD* mutant was better in medium containing aspartate as a sole nitrogen source (Figure 4H, ) or in combination with glutamine (Table S2) than in 7H9. L-asparagine is deaminated to L-aspartate by the L-asparaginase encoded by *ansA* [23,24]. Production of L-glutamate from L-aspartate could possibly be done by an aspartate aminotransferase (EC 2.6.1.1) (Figure 1). Although not assigned to EC 2.6.1.1 in the KEGG pathways for alanine, glutamate and asparagine metabolism in *M. bovis* BCG, two genes were annotated with this EC number in the PATRIC version of the KEGG pathway, namely *aspB* and BCG*_3782c* [3,25,26]. Glutamate synthesis from glutamine could be catalysed by the asparagine synthetase encoded by *asnB*, but this would require aspartate as a substrate which may explain the suppression of the *ΔgltBD* mutant’s growth when L-Gln is the sole nitrogen source [25,26]. While the other strains investigated in this study grew exponentially for a short duration when cultured in 7H9 media stripped of the nitrogen sources glutamate, ammonium sulphate and ferric ammonium citrate (the ADC supplement may be a source of trace nitrogen which may be utilized by the bacteria), growth of the *ΔgltBD* mutant (Figure 4I, ) was inhibited, suggesting that glutamate production by GOGAT may support some growth under these conditions. The importance of the GS/GOGAT system to nitrogen assimilation during limiting nitrogen conditions is well documented for several organisms including *M. tuberculosis* and *M. smegmatis* (for a review, see 27,28). Since GDH can catalyse the reductive amination of 2-oxoglutarate to produce glutamate, we hypothesised that excess ammonium would complement glutamate auxotrophy through GDH activity. However, supplementation of 7H9 with 30 mM ammonium sulphate (Figure 4J, ) did not ameliorate the poor growth of the mutant, suggesting that GDH does not produce glutamate in *M. bovis* BCG.

### *In vitro* requirement of *gdh*

**Figure 5:**
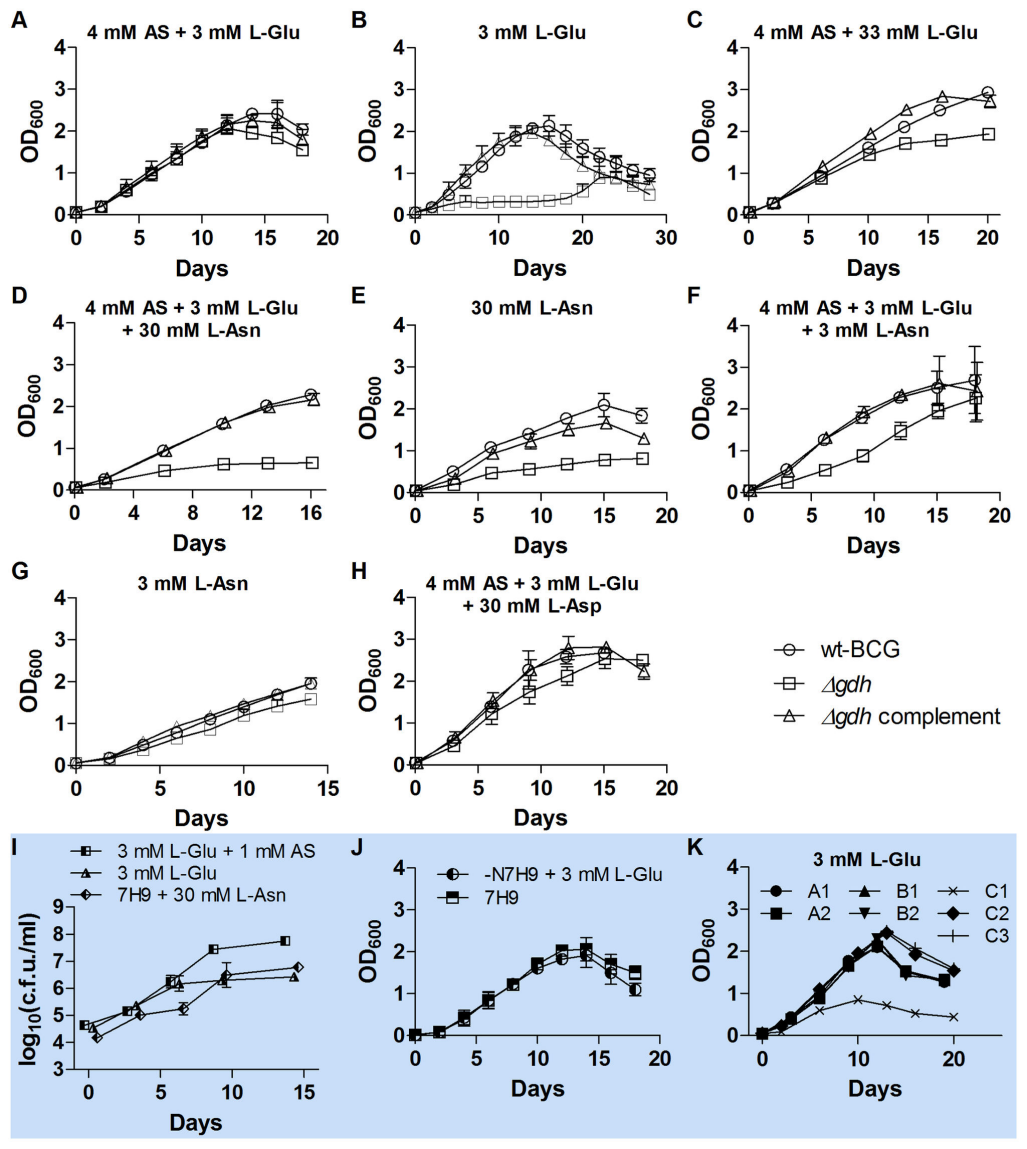
Growth of *Δgdh* in 7H9 with different nitrogen sources. Growth of wt BCG, the *Δgdh* mutant and the *Δgdh* complemented strain in (A) standard 7H9 containing approximately 4 mM ammonium sulphate (AS, (NH_4_)_2_SO_4_) and 3 mM L-Glu, (B) ‑N7H9 (nitrogen-depleted 7H9) + 3 mM L-Glu, (C) 7H9 + 30 mM L-Glu, (D) 7H9 + 30 mM L-Asn, (E) -N7H9 + 30 mM L-Asn, (F) 7H9 + 3 mM L-Asn, (G) -N7H9 + 3 mM L-Asn, or (H) 7H9 + 30 mM L-Asp. Growth of the *Δgdh* mutant in (I) ‑N7H9 + 3 mM L-Glu supplemented with increasing concentrations of AS. Cultures for cfu/ml determinations were inoculated to OD_600_ = 0.0005 (cfu/ml of approximately 10^5^). Log_10_(cfu/ml) of *Δgdh* cultured in -N7H9 + 3 mM Glu was different from log_10_(cfu/ml) of *Δgdh* cultured in -N7H9 + 3 mM Glu supplemented with 1 mM AS at day 9 and 14 (p < 0.01). Log_10_(cfu/ml) of *Δgdh* cultured in 7H9 + 30 mM L-Asn was different from log_10_(cfu/ml) of *Δgdh* cultured in -N7H9 + 3 mM Glu supplemented with 1 mM AS at day 6, 9 and 14 (p < 0.01). (J) Growth of the *Δgdh* mutant cultured in ‑N7H9 + 3 mM L-Glu for three weeks when sub-cultured in fresh 7H9 or –N7H9 + 3 mM L-Glu. Aliquots of three week old *Δgdh* mutant ‑N7H9 + 3 mM L-Glu cultures were washed once with ‑N7H9 and used to inoculate fresh 7H9 or ‑N7H9 + 3 mM L-Glu to an OD_600_ = 0.020. (K) Determination of growth of single colonies obtained from three week old *Δgdh* mutant ‑N7H9 + 3 mM L-Glu cultures in fresh –N7H9 + 3 mM L-Glu. A1 and A2 were obtained from the first growth curve experiment, B1 and B2 from the second and C1-C3 from the third. Mean OD measurements with standard deviations presented in panels A-H and J and mean log_10_(cfu/ml) with standard errors presented in panel I were calculated with growth curve data obtained from three independent experiments performed for each condition tested. In some instances error bars were smaller than the symbols used to depict the means. AS, ammonium sulphate.

Growth of the *Δgdh* mutant (Figure 5A, ) was comparable to that of wt-BCG (Figure 5A, ) in the standard 7H9 formulation, but markedly impaired when L-Glu was the sole nitrogen source (Figure 5B, ). Supplementation of 7H9 with as high concentration (30 mM) of L-Glu lead to a slight repression of growth of the *Δgdh* mutant (Figure 5C, ) in comparison to wt-BCG (Figure 5C, ) and the complemented strain (Figure 5C, ). However, growth of the *Δgdh* mutant was markedly impaired when L-Asn was present in standard 7H9 (Figure 5D, ) or –N7H9 (Figure 5E, ). Greater suppression of the *Δgdh* mutant’s growth was observed in the presence of 30 mM L-Asn (Figure 5D and E, ) than in the presence of 3 mM L-Asn (Figure 5F and G, ) and it was not suppressed by supplementation of 7H9 with 30 mM L-Asp (Figure 5H, ). Addition of 1mM ammonium sulphate to the ‑N7H9 + 3 mM L-Glu markedly improved the growth of the *Δgdh* mutant (Figure 5I, ◧). After approximately 3 weeks of culture in medium containing 3 mM L-Glu as only nitrogen source, the optical density of *Δgdh* cultures started to increase (Figure 5B, ). Contaminating micro-organisms as a source of the increase in turbidity were not detected by Ziehl-Neelsen staining as previously described [29] or by spreading out 100 μl of the cultures on blood agar plates (Becton Dickinson, USA). When aliquots of the three week old *Δgdh* mutant -N7H9 + 3 mM L-Glu cultures were washed and used to inoculate fresh ‑N7H9 + 3 mM L-Glu, immediate growth was observed (Figure 5J, ◐). Aliquots of the 3 week old ‑N7H9 + 3 mM L-Glu *Δgdh* cultures were also spread onto 7H11 plates and single colonies obtained. The single colonies were grown in 7H9 to mid-log phase and frozen stocks were prepared. Frozen stock cultures were thawed and cultured to mid-log phase and growth curve determinations were subsequently performed in ‑N7H9 + 3 mM L-Glu. Out of 7 colonies analysed, 6 colonies had similar growth profiles to that of wt-BCG (Figure 5K; A1, ; A2, ; B1, ; B2, ; C2, ; C3, ). These colonies did not have a severe growth defect in 7H9 + 30 mM L-Asn either (Figure S2). To exclude wt-BCG and the *Δgdh* complement strain as contaminating sources of the increase in turbidity observed, the colonies were analysed by PCR (Figure S3). These results suggest that a currently unknown genetic adaptation compensates for the loss of GDH activity in these colonies.

## Discussion

Despite previous reports that *gltB*, *gltD*, and *gdh*, are required for the *in vitro* growth of *M. tuberculosis* [1,11,12], we successfully generated a gene replacement mutant of the entire *gltBD* operon as well as an unmarked deletion mutant of *gdh* in *M. bovis* BCG. *M. bovis* BCG *gltB*, *gltD* and *gdh* each share 99% protein sequence identity with their homologues in *M. tuberculosis* (http://blast.ncbi.nlm.nih.gov/Blast.cgi). Our success in generating the *ΔgltBD* mutant is possibly due to our use of 7H11 agar which has a very similar formulation to 7H10 but also contains casitone. The additional casitone in 7H11 enhances growth of fastidious mycobacteria [30]. It is possible that transposon-insertion mutants of *gltB* and *gltD* were under-represented in the transposon libraries used in the transposon site hybridisation (TraSH) experiments because of an inadequate concentration of glutamate in the culture media [1,11]. We found that growth of the GOGAT-deficient mutant was only fully restored to wt-BCG levels when 10 mM L-Glu was added to 7H9 medium already containing 3.4 mM L-Glu. Interestingly, the *Δgdh* mutant grew at the same rate as wt-BCG in 7H9 and on 7H10, suggesting that *gdh* is dispensible for optimal growth in *M. bovis* BCG. This may represent a physiological difference between *M. bovis* BCG and *M. tuberculosis* which could possibly be as a result of the regions of difference in the *M. bovis* BCG genome.

We showed that GDH is important for the deamination of glutamate in *M. bovis* BCG. A physiological activity favouring the deamination of glutamate is also characteristic of other members of the L180 class of GDHs [31–34]. To our knowledge this is the first study to show that a member of the L180 class of GDHs is required for optimal growth of an organism when L-glutamate is the sole nitrogen source. Interestingly, it was previously reported that supplementation of Sauton medium containing L-Glu as nitrogen source with L-Asn resulted in an increase in extracellular Glu concentration and decreased growth of *M. tuberculosis* H37Ra [35]. We did not observe such a repressive effect for wt-BCG, but did observe repression of growth of the GDH-deficient strain by high levels of asparagine, suggesting that GDH is important in the metabolism of asparagine in *M. bovis* BCG. Activation of L180 GDH activity by L-Asn and/or L-Asp was shown for *Streptomyces clavuligerus* and *Pseudomonas aeruginosa* and L-Asp enhanced the activity of *Janthinobacterium lividum* L180 GDH by more than 1,700% [31,32,34]. These findings may suggest that glutamate catabolism by L180 GDH is promoted when asparagine/aspartate are utilized as nitrogen sources.

Our results highlight the importance of GOGAT and GDH to glutamate metabolism which may be a crucial determinant of *M. tuberculosis* survival and growth within infected cells [8]. There is no gene encoding for GOGAT in the human genome and the *M. tuberculosis* GDH is structurally and functionally different from the GDH found in humans, which may make these enzymes potential specific targets for antituberculosis drug development [33,36].

## Supporting Information

Figure S1 **Growth of wt-BCG, the *ΔgltBD* mutant, the *ΔgltBD* complement strain, *Δgdh* and the *Δgdh* complement strain on 7H10 and 7H11 agar.** Strains were cultured to early logarithmic growth phase (OD_600_ = 0.5 - 0.8) in liquid medium (see materials and methods), passed 20× through a 29GA syringe and diluted to OD_600_ = 0.0005. A dilution series was made in 7H9 and each dilution spotted (10 μl) onto the agar, which was then incubated at 37°C.
(TIF)

Figure S2 **Growth of single colonies obtained from three week old *Δgdh* mutant ‑N7H9 + 3 mM L-Glu cultures in (A) fresh –N7H9 + 3 mM L-Glu or (B) fresh 7H9 + 30 mM L-Asn.** Colonies A1 and A2 were obtained from the first growth curve experiment, B1 and B2 from the second and C2 and C3 from the third.
(TIF)

Figure S3 **Analysis of single colonies obtained from 22 day old *Δgdh* –N7H9 + 3mM L-Glu cultures.** A) Arrangement of genes in the chromosomal region of *M. bovis* BCG where *gdh* is located. B) Arrangement of genes in the *Δgdh* mutant chromosomal region where the disrupted *gdh* is located. C) Arrangement of genes in the *Δgdh* complement chromosomal region where the disrupted *gdh* is located and at the *attB* locus where pGCgdh is integrated into the chromosome. D) Gel image showing differential amplification patterns obtained when PCR was performed using the specific oligonucleotides gdhHR3R and gdhcomp10 which amplified a 1029bp product form wt-BCG template DNA (lane 1), but not from *Δgdh* mutant (lane 2), *Δgdh* complement (lane 3) or from template DNA prepared from seven single colonies obtained from 22 day old *Δgdh* –N7H9 + 3mM L-Glu cultures (lanes 4 - 10). E) Differential PCR amplification patterns obtained using the specific oligonucleotides gdhHR3R and gdhcomp6 which amplified a 3213bp product form wt-BCG template DNA (lane 1), but a 726bp product from *Δgdh* mutant (lane 2), *Δgdh* complement (lane 3) and from template DNA prepared from seven single colonies obtained from 22 day old *Δgdh* –N7H9 + 3mM L-Glu cultures (lanes 4 - 10). F) Differential PCR amplification patterns obtained using the specific oligonucleotides gdhHR8R, gdhcomp6 and gdhcomp10 which amplified a 670bp product form wt-BCG template DNA (lane 1), a 373bp product from *Δgdh* mutant template DNA (lane 2) and both a 670bp and a 373bp product from *Δgdh* complement DNA template (lane 3). This primer combination only amplified a 373bp from template DNA prepared from the seven single colonies obtained from 22 day old *Δgdh* –N7H9 + 3mM L-Glu cultures. Lane 11 (D, E and F) - negative control.
(TIFF)

Table S1 **Bacterial Strains, plasmids, and oligonucleotides used in this study.**
(DOCX)

Table S2 **Growth parameters of ΔgltBD and Δgdh mutant and complemented strains relative to wt-BCG in 7H9 and –N7H9 supplemented with different nitrogen sources.**
(DOCX)

## References

[1] Sassetti CM, Boyd DH, Rubin EJ (2003) Genes required for mycobacterial growth defined by high density mutagenesis. Mol Microbiol 48: 77–84. doi:10.1046/j.1365-2958.2003.03425.x. PubMed: 12657046. 12657046

[2] Tullius MV, Harth G, Horwitz MA (2003) Glutamine synthetase GlnA1 is essential for growth of *Mycobacterium* *tuberculosis* in human THP-1 macrophages and guinea pigs. Infect Immun 71: 3927–3936. doi:10.1128/IAI.71.7.3927-3936.2003. PubMed: 12819079. 12819079 PMC162033

[3] Gillespie JJ, Wattam AR, Cammer SA, Gabbard JL, Shukla MP et al. (2011) PATRIC: the comprehensive bacterial bioinformatics resource with a focus on human pathogenic species. Infect Immun 79: 4286–4298. doi:10.1128/IAI.00207-11. PubMed: 21896772. 21896772 PMC3257917

[4] Harth G, Horwitz MA (1997) Expression and efficient export of enzymatically active *Mycobacterium* *tuberculosis* glutamine synthetase in *Mycobacterium* *smegmatis* and evidence that the information for export is contained within the protein. J Biol Chem 272: 22728–22735. doi:10.1074/jbc.272.36.22728. PubMed: 9278431. 9278431

[5] Carroll P, Pashley CA, Parish T (2008) Functional analysis of GlnE, an essential adenylyl transferase in *Mycobacterium* *tuberculosis* . J Bacteriol 190: 4894–4902. doi:10.1128/JB.00166-08. PubMed: 18469098. 18469098 PMC2446997

[6] Parish T, Stoker NG (2000) *glnE* is an essential gene in *Mycobacterium* *tuberculosis* . J Bacteriol 182: 5715–5720. doi:10.1128/JB.182.20.5715-5720.2000. PubMed: 11004169. 11004169 PMC94692

[7] Pashley CA, Brown AC, Robertson D, Parish T (2006) Identification of the *Mycobacterium* *tuberculosis* GlnE promoter and its response to nitrogen availability. Microbiology 152: 2727–2734. doi:10.1099/mic.0.28942-0. PubMed: 16946267. 16946267

[8] Cowley S, Ko M, Pick N, Chow R, Downing KJ et al. (2004) The *Mycobacterium* *tuberculosis* protein serine/threonine kinase PknG is linked to cellular glutamate/glutamine levels and is important for growth *in* *vivo* . Mol Microbiol 52: 1691–1702. doi:10.1111/j.1365-2958.2004.04085.x. PubMed: 15186418. 15186418

[9] O’Hare HM, Durán R, Cerveñansky C, Bellinzoni M, Wehenkel AM et al. (2008) Regulation of glutamate metabolism by protein kinases in mycobacteria. Mol Microbiol 70: 1408–1423. doi:10.1111/j.1365-2958.2008.06489.x. PubMed: 19019160. 19019160

[10] Nott TJ, Kelly G, Stach L, Li J, Westcott S et al. (2009) An intramolecular switch regulates phosphoindependent FHA domain interactions in *Mycobacterium* *tuberculosis* . Sci Signal 2: ra12 PubMed: 19318624. 19318624 10.1126/scisignal.2000212

[11] Griffin JE, Gawronski JD, Dejesus MA, Ioerger TR, Akerley BJ et al. (2011) High-resolution phenotypic profiling defines genes essential for mycobacterial growth and cholesterol catabolism. PLoS Pathog 7: e1002251 PubMed: 21980284. 21980284 10.1371/journal.ppat.1002251 PMC3182942

[12] Lamichhane G, Zignol M, Blades NJ, Geiman DE, Dougherty A et al. (2003) A postgenomic method for predicting essential genes at subsaturation levels of mutagenesis: application to *Mycobacterium* *tuberculosis* . Proc Natl Acad Sci U S A 100: 7213–7218. doi:10.1073/pnas.1231432100. PubMed: 12775759. 12775759 PMC165855

[13] Webster AD, Goolamali SK (1981) BCG’osis. J R Soc Med 74: 163–165. PubMed: 6970817. 6970817 10.1177/014107688107400215 PMC1438658

[14] Sambrook J, Russell DW (2000) Molecular Cloning: A Laboratory Manual. 3rd edn.. Cold Spring Harbor Laboratory Press.

[15] Van Kessel JC, Hatfull GF (2007) Recombineering in *Mycobacterium* *tuberculosis* . Nat Methods 4: 147–152. doi:10.1038/nmeth996. PubMed: 17179933. 17179933

[16] Wards BJ, Collins DM (1996) Electroporation at elevated temperatures substantially improves transformation efficiency of slow-growing mycobacteria. FEMS Microbiol Lett 145: 101–105. doi:10.1111/j.1574-6968.1996.tb08563.x. PubMed: 8931333. 8931333

[17] Parish T, Stoker NG (1998) Electroporation of mycobacteria. Methods Mol Biol 101: 129–144. PubMed: 9921475. 9921475 10.1385/0-89603-471-2:129

[18] Machowski EE, Barichievy S, Springer B, Durbach SI, Mizrahi V (2007) *In* *vitro* analysis of rates and spectra of mutations in a polymorphic region of the *Rv0746* PE_PGRS gene of *Mycobacterium* *tuberculosis* . J Bacteriol 189: 2190–2195. doi:10.1128/JB.01647-06. PubMed: 17172340. 17172340 PMC1855714

[19] Parish T, Stoker NG (2000) Use of a flexible cassette method to generate a double unmarked *Mycobacterium* *tuberculosis* tlyA plcABC mutant by gene replacement. Microbiology 146 (8): 1969–1975. 10931901 10.1099/00221287-146-8-1969

[20] Hinds J, Mahenthiralingam E, Kempsell KE, Duncan K, Stokes RW et al. (1999) Enhanced gene replacement in mycobacteria. Microbiology 145 (3): 519–527. doi:10.1099/13500872-145-3-519. PubMed: 10217485. 10217485

[21] Warren RM, Sampson SL, Richardson M, Van Der Spuy GD, Lombard CJ et al. (2000) Mapping of IS6110 flanking regions in clinical isolates of *Mycobacterium* *tuberculosis* demonstrates genome plasticity. Mol Microbiol 37: 1405–1416. doi:10.1046/j.1365-2958.2000.02090.x. PubMed: 10998172. 10998172

[22] Chen JM, Alexander DC, Behr MA, Liu J (2003) *Mycobacterium* *bovis* BCG Vaccines Exhibit Defects in Alanine and Serine Catabolism. Infect Immun 71: 708–716. doi:10.1128/IAI.71.2.708-716.2003. PubMed: 12540549. 12540549 PMC145370

[23] Soru E, Teodorescu M, Zaharia O, Szabados J, Rudescu K (1972) L-Asparaginase from the BCG strain of *Mycobacterium* *bovis*. I. Purification and *in* *vitro* immunosuppressive properties. Can J Biochem 50: 1149–1157. doi:10.1139/o72-157. PubMed: 4629472. 4629472

[24] Jayaram HN, Ramakrishnan R, Vaidyanathan CS (1968) L-asparaginases from *Mycobacterium* *tuberculosis* strains H37Rv and H37Ra. Arch Biochem Biophys 126: 165–174. doi:10.1016/0003-9861(68)90570-5. PubMed: 4970345. 4970345

[25] Kanehisa M, Goto S, Sato Y, Furumichi M, Tanabe M (2012) KEGG for integration and interpretation of large-scale molecular data sets. Nucleic Acids Res 40: D109–D114. doi:10.1093/nar/gkr988. PubMed: 22080510. 22080510 PMC3245020

[26] Kanehisa M, Goto S (2000) KEGG: Kyoto encyclopedia of genes and genomes. Nucleic Acids Res 28: 27–30. doi:10.1093/nar/28.7.e27. PubMed: 10592173. 10592173 PMC102409

[27] Harper C, Hayward D, Wiid I, van Helden P (2008) Regulation of nitrogen metabolism in *Mycobacterium* *tuberculosis*: a comparison with mechanisms in *Corynebacterium* *glutamicum* and *Streptomyces* *coelicolor* . IUBMB Life 60: 643–650. doi:10.1002/iub.100. PubMed: 18493948. 18493948

[28] Amon J, Titgemeyer F, Burkovski A (2010) Common patterns - unique features: nitrogen metabolism and regulation in Gram-positive bacteria. FEMS Microbiol Rev 34: 588-606. PubMed: 20337720. 20337720 10.1111/j.1574-6976.2010.00216.x

[29] Bishop PJ, Neumann G (1970) The history of the Ziehl-Neelsen stain. Tubercle 51: 196–206. doi:10.1016/0041-3879(70)90073-5. PubMed: 4099679. 4099679

[30] Cohn ML, Waggoner RF, McClatchy JK (1968) The 7H11 medium for the cultivation of mycobacteria. Am Rev Respir Dis 98: 295–296. PubMed: 4299186. 4299186 10.1164/arrd.1968.98.2.295

[31] Camardella L, Di Fraia R, Antignani A, Ciardiello MA, di Prisco G et al. (2002) The Antarctic *Psychrobacter* sp. TAD1 has two cold-active glutamate dehydrogenases with different cofactor specificities. Characterisation of the NAD^+^-dependent enzyme. Comp Biochem Physiol A Mol Integr Physiol 131: 559–567. doi:10.1016/S1095-6433(01)00507-4. PubMed: 11867281. 11867281

[32] Lu CD, Abdelal AT (2001) The *gdhB* gene of *Pseudomonas* *aeruginosa* encodes an arginine-inducible NAD^+^-dependent glutamate dehydrogenase which is subject to allosteric regulation. J Bacteriol 183: 490–499. doi:10.1128/JB.183.2.490-499.2001. PubMed: 11133942. 11133942 PMC94904

[33] Miñambres B, Olivera ER, Jensen RA, Luengo JM (2000) A new class of glutamate dehydrogenases (GDH). Biochemical and genetic characterization of the first member, the AMP-requiring NAD-specific GDH of *Streptomyces* *clavuligerus* . J Biol Chem 275: 39529–39542. doi:10.1074/jbc.M005136200. PubMed: 10924516. 10924516

[34] Kawakami R, Sakuraba H, Ohshima T (2007) Gene cloning and characterization of the very large NAD-dependent l-glutamate dehydrogenase from the psychrophile *Janthinobacterium* *lividum*, isolated from cold soil. J Bacteriol 189: 5626–5633. doi:10.1128/JB.00496-07. PubMed: 17526698. 17526698 PMC1951823

[35] Lyon RH, Hall WH, Costas-Martinez C (1974) Effect of L-asparagine on growth of *Mycobacterium* *tuberculosis* and on utilization of other amino acids. J Bacteriol 117: 151–156. PubMed: 4202993. 4202993 10.1128/jb.117.1.151-156.1974 PMC246536

[36] Cheung YW, Tanner JA (2011) Targeting glutamate synthase for tuberculosis drug development. Hong Kong Med J 17 Suppl 2: 32–34. PubMed: 21368333. 21368333

