# Supplementary figures and images for: The Role of Glutamine Oxoglutarate Aminotransferase and Glutamate Dehydrogenase in Nitrogen Metabolism in Mycobacterium bovis BCG
Source: PLoS One. 2013 Dec 19;8(12):e84452. doi: 10.1371/journal.pone.0084452 (PMC3868603; doi:10.1371/journal.pone.0084452)

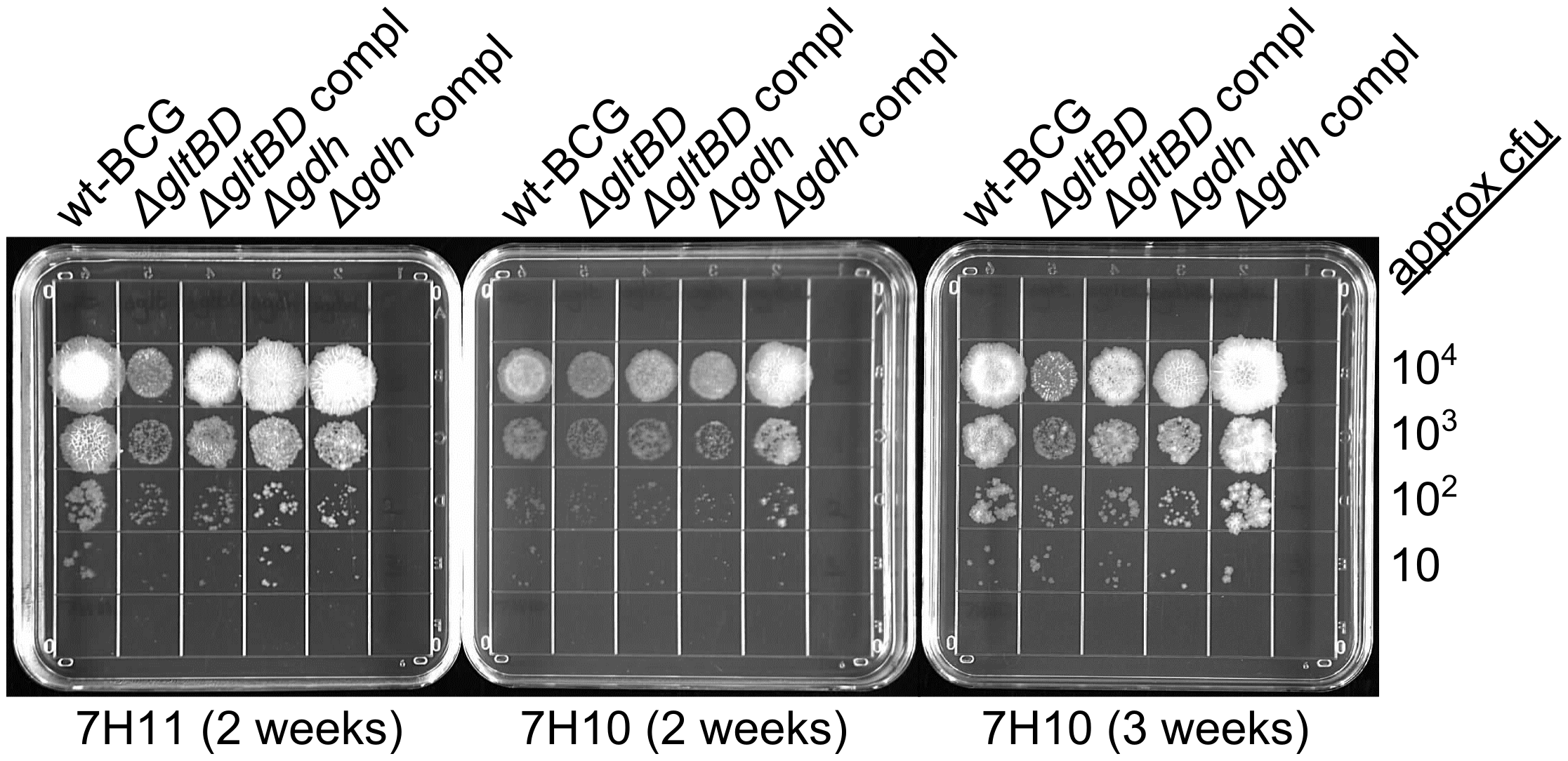

Supplement: Figure S1 — Growth of wt-BCG, the ΔgltBD mutant, the ΔgltBD complement strain, Δgdh and the Δgdh complement strain on 7H10 and 7H11 agar. Strains were cultured to early logarithmic growth phase (OD600 = 0.5 - 0.8) in liquid medium (see materials and methods), passed 20× through a 29GA syringe and diluted to OD600 = 0.0005. A dilution series was made in 7H9 and each dilution spotted (10 μl) onto the agar, which was then incubated at 37°C. (TIF) [file pone.0084452.s001.tif]

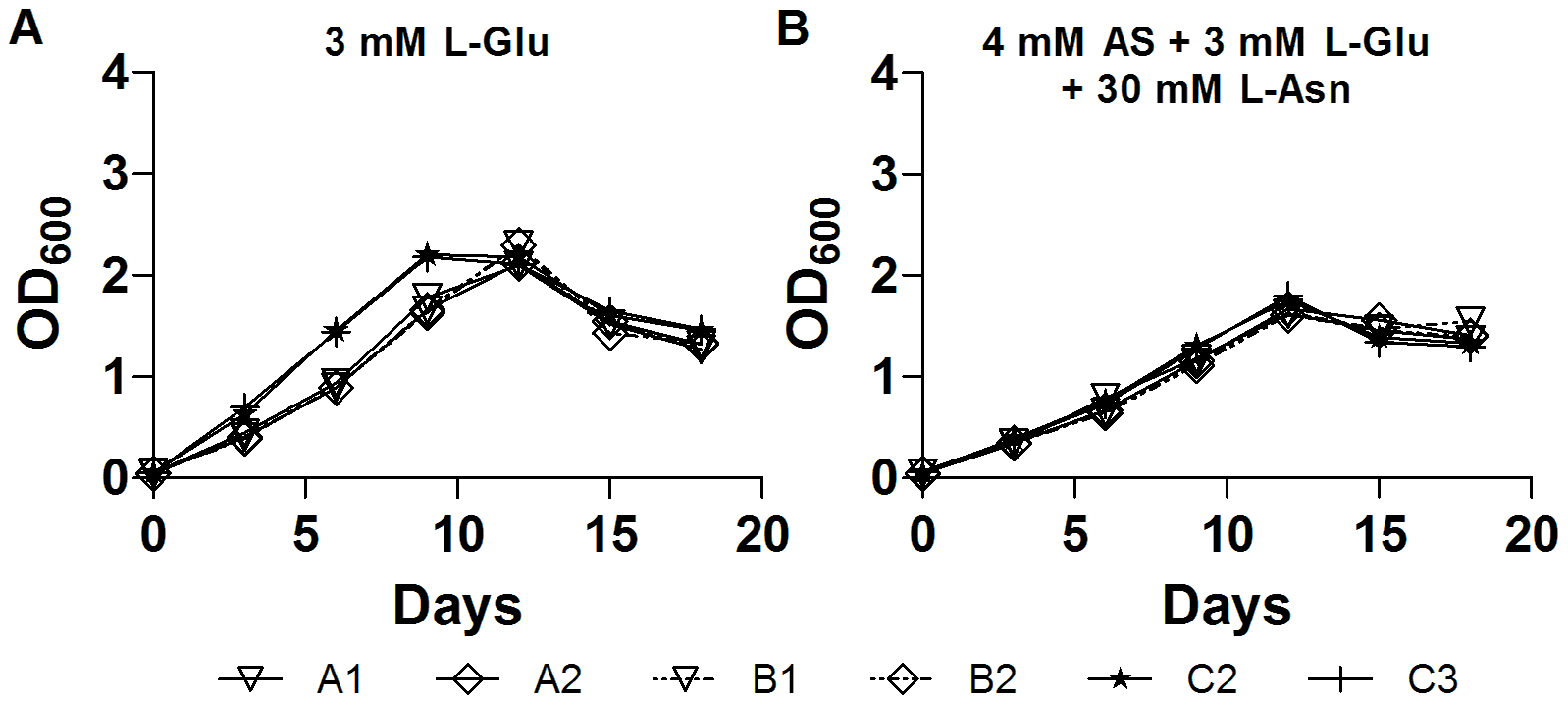

Supplement: Figure S2 — Growth of single colonies obtained from three week old Δgdh mutant ‑N7H9 + 3 mM L-Glu cultures in (A) fresh –N7H9 + 3 mM L-Glu or (B) fresh 7H9 + 30 mM L-Asn. Colonies A1 and A2 were obtained from the first growth curve experiment, B1 and B2 from the second and C2 and C3 from the third. (TIF) [file pone.0084452.s002.tif]

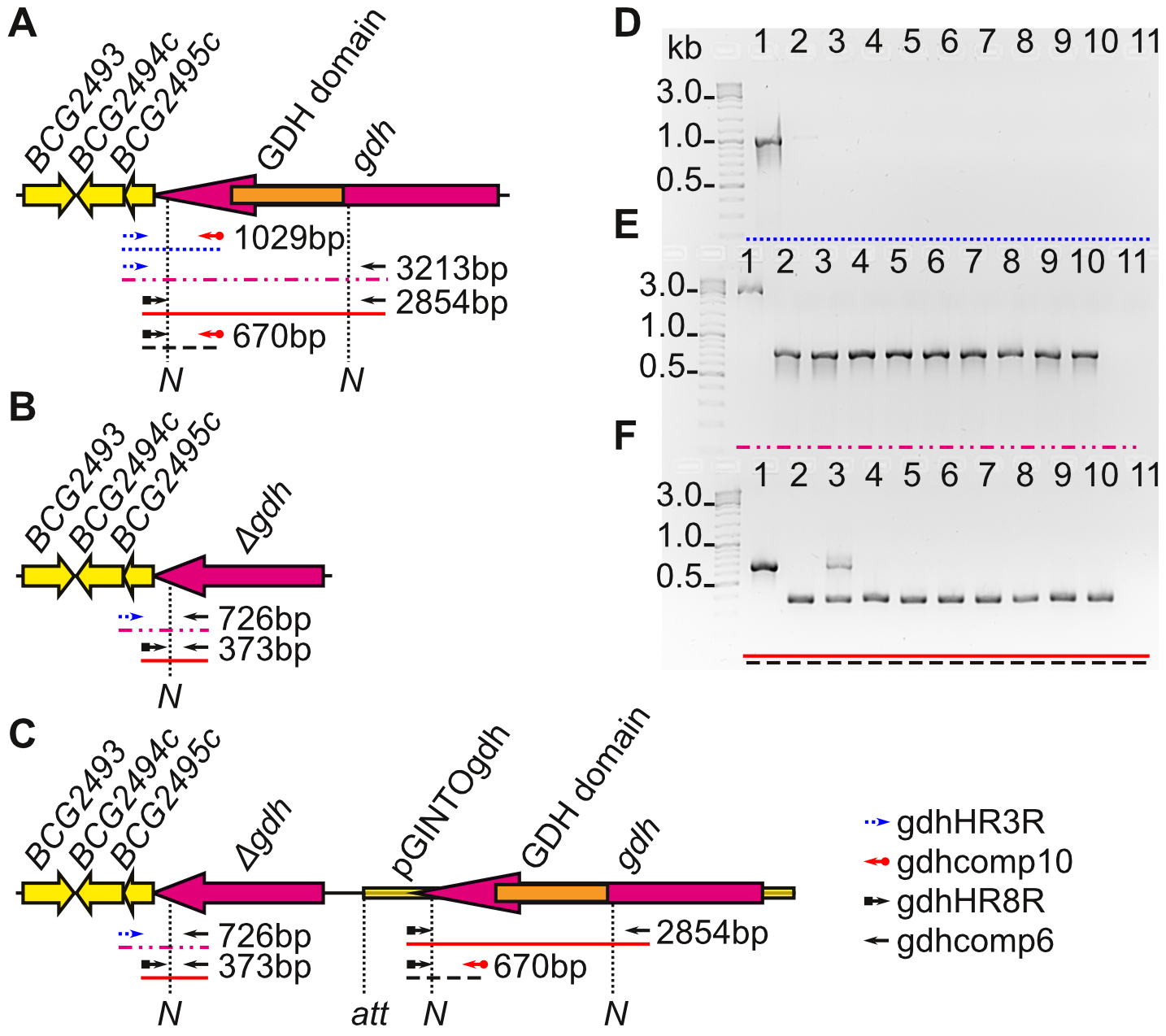

Supplement: Figure S3 — Analysis of single colonies obtained from 22 day old Δgdh –N7H9 + 3mM L-Glu cultures. A) Arrangement of genes in the chromosomal region of M. bovis BCG where gdh is located. B) Arrangement of genes in the Δgdh mutant chromosomal region where the disrupted gdh is located. C) Arrangement of genes in the Δgdh complement chromosomal region where the disrupted gdh is located and at the attB locus where pGCgdh is integrated into the chromosome. D) Gel image showing differential amplification patterns obtained when PCR was performed using the specific oligonucleotides gdhHR3R and gdhcomp10 which amplified a 1029bp product form wt-BCG template DNA (lane 1), but not from Δgdh mutant (lane 2), Δgdh complement (lane 3) or from template DNA prepared from seven single colonies obtained from 22 day old Δgdh –N7H9 + 3mM L-Glu cultures (lanes 4 - 10). E) Differential PCR amplification patterns obtained using the specific oligonucleotides gdhHR3R and gdhcomp6 which amplified a 3213bp product form wt-BCG template DNA (lane 1), but a 726bp product from Δgdh mutant (lane 2), Δgdh complement (lane 3) and from template DNA prepared from seven single colonies obtained from 22 day old Δgdh –N7H9 + 3mM L-Glu cultures (lanes 4 - 10). F) Differential PCR amplification patterns obtained using the specific oligonucleotides gdhHR8R, gdhcomp6 and gdhcomp10 which amplified a 670bp product form wt-BCG template DNA (lane 1), a 373bp product from Δgdh mutant template DNA (lane 2) and both a 670bp and a 373bp product from Δgdh complement DNA template (lane 3). This primer combination only amplified a 373bp from template DNA prepared from the seven single colonies obtained from 22 day old Δgdh –N7H9 + 3mM L-Glu cultures. Lane 11 (D, E and F) - negative control. (TIFF) [file pone.0084452.s003.tiff]
