# Supplementary material for: The Role of Glutamine Oxoglutarate Aminotransferase and Glutamate Dehydrogenase in Nitrogen Metabolism in Mycobacterium bovis BCG
Source: PLoS One. 2013 Dec 19;8(12):e84452. doi: 10.1371/journal.pone.0084452 (PMC3868603; doi:10.1371/journal.pone.0084452)
Supplement: Table S1 — Bacterial Strains, plasmids, and oligonucleotides used in this study. (DOCX) [file pone.0084452.s004.docx]

| **Table S1. Bacterial Strains, plasmids, and oligonucleotides used in this study.** | | |
| --- | --- | --- |
| **Strains/ plasmids/ oligonucleotides:** | **Description:** | **Source/reference:** |
| **Strains:** |  |  |
| ***E. coli*** |  |  |
| *DH5α* | ATCC 53868 | Laboratory collection |
| ***M. bovis* BCG** |  |  |
| wt-BCG | Wild type *M. bovis* BCG str. *Pasteur 1743P2*, progenitor strain of all other *M. bovis* BCG strains in the study | Laboratory collection |
| wt-BCG pJV53 | wt*-*BCG strain carrying pJV53; recombineering proficient strain used to make allelic replacement mutants | This study |
| *ΔgltBD*::*hyg* (referred to as *ΔgltBD*) | gene replacement of *gltBD* operon with a hygromycin cassette, carries recombineering plasmid pJV53, *hyg^R^*, *kan^R^* | This study |
| *ΔgltBD attB*::pGCgltBD | complemented strain of *ΔgltBD*::*hyg*, carries pGCgltBD and pJV53, *hyg^R^*, *kan^R^*, *gent^R^* | This study |
| *Δgdh* | *Δgdh*, deletion of 2487bp *NruI* fragment spanning the GDH domain within the *gdh* gene. | This study |
| *Δgdh attB*::pGCgdh | complemented strain of *Δgdh*, carries pGCgdh, *gent^R^* | This study |
| **Plasmids:** |  |  |
| pJV53 | Expresses the che9c mycobacteriophage enzymes gp60 and gp61 under acetamide induction, *oriC*, *oriM*, *kan^R^* | Van Kessel *et al.*, 2007 |
| pGEM-T easy | *E. coli* vector for cloning PCR products, *amp^R^* | Promega |
| pMNFhyg | Hygromycin cassette TA-cloned into the pGEM-T Easy vector (Promega), *hyg^R^* | Newton-Foot *et al.*, unpublished data |
| pMNFhygU | UgltBD PCR product TA cloned into pGEM-T Easy, excised with *SphI*-*NcoI* and ligated to pMNFhyg which was linearised with *SphI* and *NcoI*. | This study |
| pAVΔgltBD | DgltBD PCR product TA cloned into pGEM-T Easy, excised with *SpeI*-*PstI* and ligated to pMNFgltU which was linearised with *SpeI* and *PstI*. | This study |
| pGINTO | *integrase*, *attP*, *gent^R^* | Machowski *et al.*, 2007 |
| pGCgltBD | 6662 bp CgltBD PCR product blunt-end cloned into pGINTO linearized with *ScaI* | This study |
| p2Nil | kan^R^, oriE | Parish *et al.*, 2000 |
| pGOAL17 | amp^R^, lacZ, sacB, oriE | Parish *et al.*, 2000 |
| pGEMgdh | 4624 bp gdh1 PCR amplicon T/A cloned into pGEM-T Easy | This study |
| pGEMΔgdh | 2487 bp GDH domain spanning NruI fragment deleted from pGEMgdh | This study |
| p2NilΔgdh | *KpnI* fragment from pGEMΔgdh containing *Δgdh* sequence cloned into the single *KpnI* site of p2Nil. | This study |
| pAVΔgdh | *PacI* fragment from pGOAL17 containing the *sacB* and *lacZ* cassettes cloned into the single *PacI* site of p2NilΔgdh. | This study |
| pGCgdh | 5439 bp Cgdh PCR product blunt-end cloned into pGINTO linearized with *ScaI* | This study |
| **Oligonucleotides:** |  |  |
| UgltBDF | 5’‑GCATGCCCGACCAATATCGTCCC‑3’, *SphI* recognition sequence on 5’ end | This study |
| UgltBDR | 5’‑CCTAGGCGATAGGCTGTCCGTCAA‑3’, *NcoI* recognition sequence on 5’ end | This study |
| DgltBDF | 5’‑ACTAGTAGCTCCGAGGTGTCTAATG‑3’, *SpeI* recognition sequence on 5’ end | This study |
| DgltBDR | 5’‑CTGCAGATTTCGCCGAGGATGA‑3’, *PstI* recognition sequence on 5’ end | This study |
| CgltBDF | 5’‑P‑CCCTTTTAGTATCGCGCAAA‑‑3’ | This study |
| CgltBDR | 5’‑P‑GAAGCCCACATCGACTCATT‑3’ | This study |
| gdhF | 5’‑GGTACCGCTTCGTCGGGCTCTTC-3’, *KpnI* recognition site on 5’ end. | This study |
| gdhR | 5’-GGTACCAGGCATCCGTTGTGGC-3’, *KpnI* recognition site on 5’ end. | This study |
| PgdhF | 5’‑TGCTGCTCCGTGCCTAC‑3’ | This study |
| PgdhR | 5’‑ACGAACCCGACGCTCA‑3’ | This study |
| CgdhF | 5’‑P‑GTCTCGGACGGTCTGGACTA‑3’ | This study |
| CgdhR | 5’‑P‑GCTCACCCCGAGATTCC‑3’ | This study |
| gdh3R | 5’‑TCATCAGCTCACGGGCCCCCGGTA‑3’ | This study |
| Cgdh10 | 5’‑ CGGAATGGAACGATCTGGT‑3’ | This study |
| Cgdh6 | 5’‑ GTTCAACCCGGAGACTGTGT‑3’ | This study |
