# Supplementary material for: The Role of Glutamine Oxoglutarate Aminotransferase and Glutamate Dehydrogenase in Nitrogen Metabolism in Mycobacterium bovis BCG
Source: PLoS One. 2013 Dec 19;8(12):e84452. doi: 10.1371/journal.pone.0084452 (PMC3868603; doi:10.1371/journal.pone.0084452)
Supplement: Table S2 — Growth parameters of ΔgltBD and Δgdh mutant and complemented strains relative to wt-BCG in 7H9 and –N7H9 supplemented with different nitrogen sources. (DOCX) [file pone.0084452.s005.docx]

| **Table S2. Growth parameters of *ΔgltBD* and *Δgdh* mutant and complemented strains relative to wt-BCG in 7H9 and –N7H9 supplemented with different nitrogen sources.** | | | | | | | | | | | | | | | | |
| --- | --- | --- | --- | --- | --- | --- | --- | --- | --- | --- | --- | --- | --- | --- | --- | --- |
|  | ***ΔgltBD*** | | | | ***ΔgltBD* complement** | | | | ***Δgdh*** | | | | ***Δgdh* complement** | | | |
|  | **A** | | **R** | | **A** | | **R** | | **A** | | **R** | | **A** | | **R** | |
| **7H9 (≈4 mM (NH_4_)_2_SO_4_ + ≈3 mM L-Glu)** |  |  |  |  |  |  |  |  |  |  |  |  |  |  |  |  |
| unmodified | 0.25 ± 0.05 | *** | 0.65 ± 0.31 | ns | 1.03 ± 0.09 | ns | 1.01 ± 0.15 | ns | 0.85 ± 0.11 | * | 0.99 ± 0.31 | ns | 0.95 ± 0.06 | ns | 1.05 ± 0.12 | ns |
| + 30 mM L-Asn | 0.63 ± 0.18 | ** | 0.92 ± 0.16 | ns | 0.81 ± 0.32 | ns | 1.32 ± 0.26 | ** | 0.25 ± 0.01 | *** | 0.43 ± 0.12 | *** | 0.83 ± 0.10 | ns | 1.11 ± 0.13 | ns |
| + 3 mM L-Asn | 0.40 ± 0.14 | ** | 0.84 ± 0.24 | ns | 0.93 ± 0.22 | ns | 1.27 ± 0.21 | ns | 0.77 ± 0.13 | ns | 0.86 ± 0.26 | ns | 0.92 ± 0.11 | ns | 1.20 ± 0.09 | ns |
| + 30 mM (NH_4_)_2_SO_4_ | 0.29 ± 0.05 | *** | 0.66 ± 0.19 | * | 1.01 ± 0.23 | ns | 0.96 ± 0.15 | ns | 0.88 ± 0.24 | ns | 1.14 ± 0.02 | ns | 0.95 ± 0.16 | ns | 1.10 ± 0.09 | ns |
| + 3 mM L-Asp | 0.56 ± 0.09 | ** | 0.54 ± 0.05 | *** | 0.97 ± 0.09 | ns | 1.05 ± 0.05 | ns | 1.08 ± 0.39 | ns | 0.81 ± 0.23 | ns | 1.11 ± 0.15 | ns | 1.15 ± 0.13 | ns |
| + 30 mM L-Asp | 0.91 ± 0.53 | ns | 0.77 ± 0.25 | ns | 0.75 ± 0.23 | ns | 1.12 ± 0.30 | ns | 0.77 ± 0.21 | ns | 0.81 ± 0.08 | ns | 0.78 ± 0.35 | ns | 1.21 ± 0.40 | ns |
| + 30 mM L-Glu | 0.88 ± 0.08 | ns | 1.12 ± 0.17 | ns | 0.86 ± 0.37 | ns | 1.21 ± 0.32 | ns | 0.59 ± 0.03 | ** | 0.96 ± 0.13 | ns | 0.94 ± 0.08 | ns | 1.31 ± 0.06 | ns |
| + 10 mM L-Glu | 0.98 ± 0.10 | ns | 1.01 ± 0.11 | ns | 1.01 ± 0.06 | ns | 1.01 ± 0.16 | ns | 0.65 ± 0.23 | ** | 0.94 ± 0.25 | ns | 0.87 ± 0.16 | ns | 1.23 ± 0.05 | ns |
| + 30 mM L-Gln | nd |  | nd |  | nd |  | nd |  | 0.98 ± 0.15 | ns | 1.25 ± 0.10 | * | 0.98 ± 0.15 | ns | 1.18 ± 0.11 | ns |
| **-N7H9** |  |  |  |  |  |  |  |  |  |  |  |  |  |  |  |  |
| unmodified | 0.31 ± 0.08 | ** | 0.57 ± 0.40 | ns | 1.05 ± 0.11 | ns | 1.18 ± 0.25 | ns | 0.94 ± 0.02 | ns | 1.08 ± 0.51 | ns | 1.13 ± 0.13 | ns | 1.02 ± 0.22 | ns |
| + 3 mML-Glu | 0.30 ± 0.13 | *** | 0.61 ± 0.39 | ns | 0.97 ± 0.15 | ns | 1.01 ± 0.29 | ns | 0.19 ± 0.11 | *** | 0.31 ± 0.38 | ** | 0.91 ± 0.03 | ns | 1.14 ± 0.32 | ns |
| + 30 mM L-Asn + 3 mM L-Glu | nd |  | nd |  | nd |  | nd |  | 0.42 ± 0.08 | *** | 0.39 ± 0.15 | ** | 0.79 ± 0.04 | ** | 1.03 ± 0.25 | ns |
| + 30 mM L-Asn + 4 mM (NH_4_)_2_SO_4_ | nd |  | nd |  | nd |  | nd |  | 0.41 ± 0.02 | *** | 0.43 ± 0.08 | ** | 0.78 ± 0.01 | *** | 1.03 ± 0.23 | ns |
| + 3 mM L-Asp + 3 mM L-Glu | nd |  | nd |  | nd |  | nd |  | 0.65 ± 0.16 | ** | 0.29 ± 0.13 | ** | 0.82 ± 0.05 | ns | 1.18 ± 0.05 | ns |
| + 4 mM (NH_4_)_2_SO_4_ | 0.05 ± 0.03 | *** | 0.08 ± 0.01 | *** | 1.03 ± 0.09 | ns | 0.96 ± 0.16 | ns | 0.93 ± 0.11 | ns | 0.92 ± 0.22 | ns | 0.94 ± 0.16 | ns | 0.91 ± 0.13 | ns |
| + 30 mM L-Asn | 0.56 ± 0.06 | * | 0.81 ± 0.06 | ns | 1.49 ± 0.74 | * | 1.05 ± 0.13 | ns | 0.42 ± 0.09 | ** | 0.38 ± 0.18 | *** | 0.75 ± 0.05 | ns | 1.02 ± 0.34 | ns |
| + 3 mM L-Gln | 0.15 ± 0.04 | *** | 0.18 ± 0.02 | *** | 1.11 ± 0.24 | ns | 0.97 ± 0.28 | ns | 0.95 ± 0.07 | ns | 1.09 ± 0.07 | ns | 0.99 ± 0.06 | ns | 0.97 ± 0.13 | ns |
| + 3 mM L-Asp | 0.74 ± 0.29 | ns | 0.67 ± 0.36 | ns | 1.09 ± 0.20 | ns | 1.10 ± 0.47 | ns | 0.74 ± 0.32 | ns | 0.32 ± 0.04 | *** | 0.90 ± 0.06 | ns | 1.20 ± 0.24 | ns |
| + 3 mM L-Asn | 0.25 ± 0.07 | *** | 0.74 ± 0.05 | ns | 1.17 ± 0.31 | ns | 0.95 ± 0.22 | ns | 0.77 ± 0.15 | * | 0.88 ± 0.23 | ns | 1.07 ± 0.07 | ns | 0.95 ± 0.17 | ns |
| + 3 mM L-Asn + 3 mM L-Asp | nd |  | nd |  | nd |  | nd |  | 0.85 ± 0.08 | ns | 0.78 ± 0.08 | ns | 0.91 ± 0.13 | ns | 1.13 ± 0.08 | ns |
| + 3 mM L-Asn + 3 mM L-Glu | nd |  | nd |  | nd |  | nd |  | 0.83 ± 0.05 | * | 0.83 ± 0.19 | ns | 0.99 ± 0.15 | ns | 1.08 ± 0.09 | ns |
| + 3 mM L-Asn + 4 mM (NH_4_)_2_SO_4_ | nd |  | nd |  | nd |  | nd |  | 0.83 ± 0.05 | ** | 0.94 ± 0.07 | ns | 0.94 ± 0.08 | ns | 1.16 ± 0.19 | ns |
| + 3 mM L-Asp + 3 mM L-Gln | 1.10 ± 0.26 | ns | 0.67 ± 0.16 | * | 1.17 ± 0.31 | ns | 0.93 ± 0.26 | ns | nd |  | nd |  | nd |  | nd |  |
| + 3 mM L-Asn + 3 mM L-Gln | nd |  | nd |  | nd |  | nd |  | 0.92 ± 0.08 | ns | 1.01 ± 0.18 | ns | 1.09 ± 0.06 | * | 1.21 ± 0.13 | ns |
| + 3 mM L-Asn + 3 mM L-Ala | nd |  | nd |  | nd |  | nd |  | 1.01 ± 0.07 | ns | 1.10 ± 0.15 | ns | 0.97 ± 0.05 | ns | 0.97 ± 0.10 | ns |
| The growth parameters maximal growth achieved (A) and maximal growth rate (R) were estimated by modelling growth curve data with the Logistic or Gompertz sigmoidal growth functions [1]. Akaike’s Information Criteria was used to select the model which was most likely to have generated the growth curve data in each instance. Data presented are the mean A or R growth parameters and their associated standard errors calculated from three independent growth curve experiments. Comparisons made between the strains listed above and wt-BCG were analysed by one-way ANOVA with Bonferroni post-testing. 🞱 - p < 0.05, 🞱🞱 - p < 0.01, 🞱🞱🞱 - p < 0.001, ns - non-significant, nd – no data | | | | | | | | | | | | | | | | |

1. Zwietering MH, Jongenburger I, Rombouts FM, van ’t Riet K (1990) Modeling of the Bacterial Growth Curve. Appl Environ Microbiol 56: 1875–1881.
